# Estrogen regulation of TRPM8 expression in breast cancer cells

**DOI:** 10.1186/1471-2407-10-212

**Published:** 2010-05-19

**Authors:** Dechen Chodon, Arnaud Guilbert, Isabelle Dhennin-Duthille, Mathieu Gautier, Marie-Sophie Telliez, Henri Sevestre, Halima Ouadid-Ahidouch

**Affiliations:** 1 Laboratoire de Physiologie Cellulaire et Moléculaire, JE "2530: Canaux ioniques dans le cancer du sein", Faculté des Sciences, Université Picardie Jules Vernes, 33 rue Saint Leu, 80000, Amiens, France; 2 Service d'Anatomie Pathologique, CHU Nord, Amiens, France

## Abstract

**Background:**

The calcium-permeable cation channel TRPM8 (melastatin-related transient receptor potential member 8) is over-expressed in several cancers. The present study aimed at investigating the expression, function and potential regulation of TRPM8 channels by ER alpha (estrogen receptor alpha) in breast cancer.

**Methods:**

RT-PCR, Western blot, immuno-histochemical, and siRNA techniques were used to investigate TRPM8 expression, its regulation by estrogen receptors, and its expression in breast tissue. To investigate the channel activity in MCF-7 cells, we used the whole cell patch clamp and the calcium imaging techniques.

**Results:**

TRPM8 channels are expressed at both mRNA and protein levels in the breast cancer cell line MCF-7. Bath application of the potent TRPM8 agonist Icilin (20 μM) induced a strong outwardly rectifying current at depolarizing potentials, which is associated with an elevation of cytosolic calcium concentration, consistent with established TRPM8 channel properties. RT-PCR experiments revealed a decrease in TRPM8 mRNA expression following steroid deprivation for 48 and 72 hours. In steroid deprived medium, addition of 17-beta-estradiol (E_2_, 10 nM) increased both TRPM8 mRNA expression and the number of cells which respond to Icilin, but failed to affect the Ca^2+ ^entry amplitude. Moreover, silencing ERα mRNA expression with small interfering RNA reduced the expression of TRPM8. Immuno-histochemical examination of the expression of TRPM8 channels in human breast tissues revealed an over-expression of TRPM8 in breast adenocarcinomas, which is correlated with estrogen receptor positive (ER^+^) status of the tumours.

**Conclusion:**

Taken together, these results show that TRPM8 channels are expressed and functional in breast cancer and that their expression is regulated by ER alpha.

## Background

Breast cancer is the most common form of cancer in women in industrialized countries. Clinical and experimental data have revealed that female steroid hormones play an essential role in the development of sporadic breast cancer [1]. For example, the steroid hormone 17-β-estradiol (E_2_) is involved in the regulation of growth and differentiation of malignant breast epithelial cells [2]. The predominant biological effect of E_2 _is mediated through its interaction with two intracellular estrogen receptors (ERα and ERβ), ERα being strongly expressed in 80% of breast cancers [2-4]. ERs are ligand-dependent transcription factors controlled by E_2_, and they regulate the expression of many genes [1-5] including potassium [6], calcium [7,8] and TRP (transient receptor potential) channels [9,10].

Recently, TRP channels have emerged as new channels implicated in carcinogenesis [11-14]. In our laboratory, we have previously shown that TRPC6 (canonical-related TRP member 6) channels are over-expressed and functional in breast cancer [15]. More recently, we have shown that TRPM7 (melastatin-related TRP member 7) is involved in breast cancer cell proliferation [16]. In addition, TRPM8 (melastatin-related TRP member 8) channel is found to be over-expressed in several primary tumours including colon, lung, skin, and prostate cancer [17]. TRPM8 channel is a Ca^2+^-permeable cation channel which is stimulated by temperatures below 28°C and by the cooling agents Menthol and Icilin [18,19].

It is well established that TRPM8 channel expression is regulated by androgens. Indeed, the androgen regulation of TRPM8 expression was reported in prostate cancer and putative androgen receptor response elements were identified in the TRPM8 gene [20-22]. Therefore, TRPM8 channels can be considered as a valuable prognostic marker in prostate cancer [23]. However, in breast cancer, TRPM8 function and regulation by E_2 _are unknown. Only a single report has mentioned the over-expression of TRPM8 mRNA in breast cancer [17].

It is now well established that breast cancer cell line MCF-7 expresses E_2 _receptors (ER^+^), and that 17-β-estradiol increases its proliferation [24]. In the present study, we examined the expression and function of TRPM8 in the ER^+ ^human breast cancer cell line MCF-7. We also determined whether TRPM8 mRNA expression was regulated by estrogens. Finally, we investigated whether TRPM8 is over-expressed in human breast cancer tissues regarding their ER status.

## Methods

### Cell culture and steroid depletion procedure

MCF-7 cells were routinely cultured in Dulbecco's Modified Eagle's Medium (Lonza, Belgium) including 4.5 g/L glucose and L-glutamine, supplemented with 5% fetal calf serum (FCS), and maintained at 37°C in a humidified atmosphere with 5% CO_2_. For steroid depletion experiments, MCF-7 cells were seeded in 60-mm Petri dishes at a density of 6.10^5 ^cells/dish and grown for 48 h. Then, cells were grown in a medium in which the FCS was replaced with starvation medium (0FCS) or steroid-free medium (5% dextran-coated charcoal-treated FCS (DCCFCS) for 24, 48 and 72 h prior to incubation with E_2 _(10 nM) for 24 and 48 h E_2 _(Sigma, France) was dissolved in ethanol, and final E_2 _concentration was obtained by appropriate dilution. The dilution factor was < 1/100000.

### Electrophysiological recording

MCF-7 cells were seeded in 35-mm Petri dishes at a confluence of 8.10^4 ^cells/dish. They were grown at 37°C in a humidified atmosphere of air/CO_2 _for further 48 h before electrophysiological recordings. Dishes with attached cells were transferred to a continually perfused recording chamber and TRPM8 activity was recorded using the conventional patch clamp technique in the whole cell configuration. Patch pipettes were made using haematocrit capillaries (Hirschmann-Laborgerate, Germany. Patch pipettes of 3-5 MΩ were filled with (in mM): CsCl 145, NaCl 8, MgCl_2 _2, EGTA 10, and HEPES 10 (pH was adjusted to 7.2 using CsOH). External solution for patch-clamp recordings contained (in mM): NaCl 140, KCl 5, CaCl_2 _2, MgCl_2 _2, HEPES 10, Glucose 5, and TEA-Cl 5 (pH was adjusted to 7.4 using NaOH). Activation of TRPM8 currents was achieved by external application of 20 μM Icilin. Current-Voltage relationship was obtained by linear 100 ms ramps from -100 mV to +100 mV from a holding potential of -40 mV. Signals were captured using a Digidata 1200 converter and they were analysed using an Axopatch 200B in combination with pClamp 9 software (All from Molecular Devices, Sunnyvale, CA, USA). Traces were filtered at 5 kHz and digitized at 10 kHz. Analyses were made using Clampfit 9 (Molecular Devices, Sunnyvale, CA, USA) and Microcal Origin 8.0 software (Microcal Software, Northampton, MA, USA). Experiments were performed at room temperature (21°C).

### Calcium imaging

Calcium imaging experiments, using Fura-2, have been carried out as previously described [25]. The extracellular solution contained (in mM): 145 NaCl, 5 KCl, 5 CaCl_2_, 2 MgCl_2_, 10 HEPES, and 5 glucose (pH adjusted to 7.4 by NaOH). Icilin and thapsigargin (TG, Sigma Aldrich, France) were dissolved in DMSO. The dilution factor was < 1/1000.

### Reverse transcription and semi-quantitative PCR

Total RNA extraction and reverse transcription of RNA was carried out as previously described [15]. Sense and antisense PCR primers specific to TRPM8 channels (sense: 5'-TCTACGAGCCCTACCTG-3', antisense: 5'-CACCGTGTAGCCAAAC-3'), ERα (sense: 5'-AGGTGTACCTGGACAGCAGCAAG-3', antisense: 5'-TCTAGAAGGTGGACCTGATCATG-3'), and β-actin (sense: 5'-CAGAGCAAGAGAGGCATCCT-3', antisense: 5'-ACGTACATGGCTGGGGTG-3') were used. PCR reactions were carried out on a iCycler thermal cycler (Biorad, France) using the following parameters: denaturation at 94°C for 30 s, annealing at 58°C (TRPM8 and β-actin) or 60°C (ERα) for 30 s, and extension at 72°C for 40 s. 23 cycles for β-actin and 40 cycles for TRPM8 and ERα primers were performed, followed by an extension at 72°C for 5 min. PCR products were analyzed by gel electrophoresis and visualized by ethidium bromide staining. PCR products were quantified using Quantity One software (Biorad, France) and results are expressed as the ratio of ERα or TRPM8 on β-actin referent gene.

### Small Interfering (si) RNA cell transfection

MCF-7 cells were transfected as previously described [26] using 2 μg ERα siRNA (SiERα) on target plus smart pool L-003401-00-0005 human ESR1 (Dharmacon, USA). Control experiments were performed by transfecting 2 μg siRNA (SiControl) which does not target any known gene (D-001210-01-20, Dharmacon, USA).

### Immunoprecipitation and Western Blotting

MCF-7 cells were lysed in RIPA buffer (Triton X-100 1%, sodium deoxycholate 1%, NaCl 150 mM, Tris HCl 50 mM pH 7.4, Sigma P8340 inhibitors cocktail, EDTA 2 mM, sodium orthovanadate 0.5 mM). Human colon cancer tissue proteins were extracted in the WCE buffer (Whole Cell Extract: NaCl 150 mM, Tris HCl 50 mM pH7.5, NP40 1%, Sigma P8340 inhibitors cocktail, SDS 0.1% and sodium orthovanadate 1 mM) using a Polytron homogenizer (PRO-200, Fisher Bioblock Scientific). Equal amount of each protein sample (30 μg) were separated by electrophoresis on SDS-PAGE and blotted onto nitrocellulose membrane (GE Healthcare). Blots were incubated as indicated with antibodies raised against TRPM8 (Abcam, 1/500) and β-actin (Santa Cruz, 1/2000) proteins. The blots were developed with the enhanced chemiluminescence (ECL) system (Bio-rad) using specific peroxidase-conjugated anti-IgG antibodies.

Immunoprecipitation experiments were performed on 1 mg of protein sample from MCF-7 cells. Lysates were precleared 30 min with 50 μl of protein G- and A-agarose beads. Supernatants were incubated overnight at 4°C with 1 μg of TRPM8 antibody (Abcam) and then incubated with protein G- and A-agarose beads for 30 min. Beads were washed three times with RIPA buffer and resuspended in SDS loading buffer before electrophoresis.

### Immuno-histochemistry

Tumour and non-tumour tissue samples were selected by pathologists from fresh specimens and embedded in paraffin until analysis. Surgical consent forms were signed by the patients to allow the use of a portion of the cancerous tissue for research purposes. Samples were considered as ER^+ ^when ERα was expressed. ERα immuno-histochemical staining (ER^+ ^or ER^-^) and tumour grade were evaluated by the pathologists in routine examination. The Scarff, Bloom and Richardson (SBR) histopronostic grading system is based on cell differentiation, nuclear polymorphism and mitotic activity: Grade I (well-differentiated), Grade II (moderately-differentiated) and Grade III (poorly-differentiated).

TRPM8 immuno-histochemical studies were performed as previously described [15] using the indirect immuno-peroxidase staining technique on paraffin-embedded material with a Ventana ES automatic analyzer (Ventana Medical Systems) and with a haematoxylin counterstain. Briefly, after blocking the endogenous peroxidase by the I-View Inhibitor (Ventana), sections were stained with an anti-TRPM8 antibody (Abcam, 1/1500), incubated with biotinylated anti-rabbit IgG (I-View Biotin Ig, Ventana) and exposed to streptavidine-peroxidase complex (I-View SA-HRP, Ventana). DAB/H_2_O_2 _was used as chromogen and the slides were then examined under optical microscopy. Micrograph acquisition was performed by a camera connected to a Zeiss microscope equipped with a x 20 objective lens. Immuno-staining levels in the tumour tissue were determined by subjective visual scoring of the brown stain, and compared to the non tumoral tissue. Scoring levels were: 0 = absence of staining; 1 = weak staining intensity (equal to normal tissue); 2 = moderate; 3 = strong staining intensity. For the quantitative analysis, we report the percentage of cases presenting an over-expression of TRPM8 (scores 2 and 3).

All experiments on human tissues were approved by the Comité Consultatif de Protection des Personnes dans la Recherche Biomédicale de Picardie (CCPPRB), Amiens, France.

### Statistics

Results are expressed as means ± SEM. Unpaired Student *t-*tests were used for mRNA expression levels analysis. *χ2 *tests were used to estimate the significance between the effects of Icilin on the Ca^2+ ^signal in E_2 _treated cells, and also to estimate the correlation between TRPM8 over-expression and adenocarcinomas ER status. Differences were considered significant when p < 0.05.

## Results

### Evidence of functional TRPM8 channels in MCF-7 breast cancer cell line

**Figure 1:**
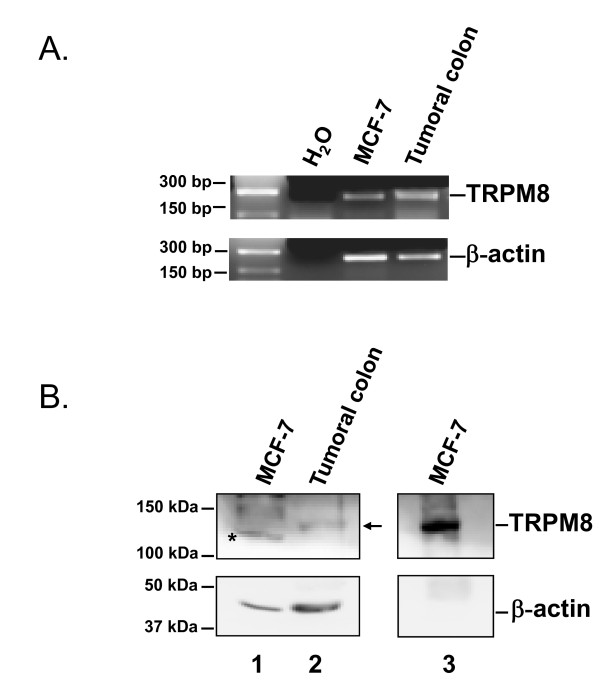
**Analysis of TRPM8 mRNA and protein expression in MCF-7 cells. **(A) RT-PCR analysis of human TRPM8 transcripts expression in MCF-7 cells on three independent cell lysates. Human colon extract was used as positive control for the detection of TRPM8. (B) Western blot analysis of TRPM8 proteins were performed on total lysate (lanes 1-2) of two independent cell extracts, or after immunoprecipitation experiment (lane 3). The arrow indicates a TRPM8 band, and (*) a non specific band.

In order to examine whether TRPM8 channels are expressed in MCF-7 cells, RT-PCR and Western Blotting experiments were carried out (Fig. 1). We detected TRPM8 mRNA in MCF-7 cells, and in human colon cancer tissue which was used as a positive control (Fig. 1A). TRPM8 proteins were also observed in human colon cancer tissue and in a lesser extent in MCF-7 cells (Fig. 1B, lanes 1-2). Immunoprecipitation experiments using the same Abcam antibody confirmed the presence of TRPM8 proteins in MCF-7 cells (Fig. 1B, lane 3).

**Figure 2:**
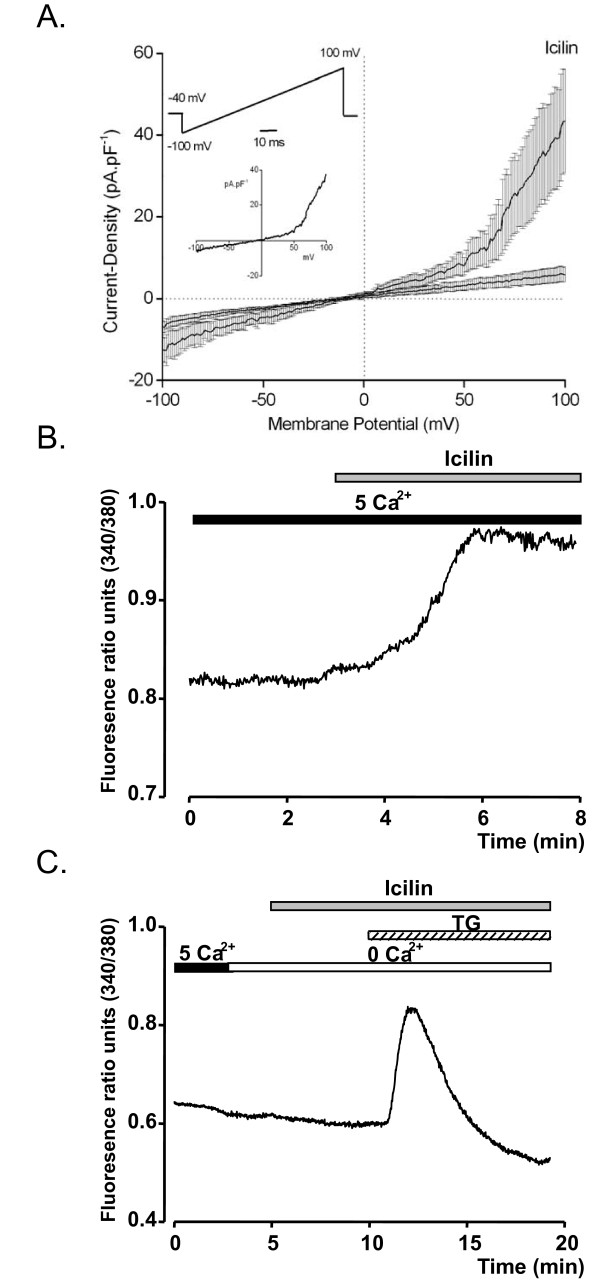
**Icilin activated functional plasma membrane TRPM8 channel in MCF-7 breast cancer cell line. **(A) Averaged I-V relationship of membrane current-densities in MCF-7 cells before and after application of Icilin (20 μM) in the bath (n = 4). Upper panel represents the patch-clamp protocol and lower panel the Icilin-activated current. Whole cell currents were elicited by a 100 ms linear ramp from -100 mV to +100 mV from a holding potential of -40 mV. (B) Average time-course of [Ca^2+^]_c _variation measured as fluorescence at 340/380 nm ratio (n = 11). (C) Average time-course of [Ca^2+^]_c _changes (n = 20) during perfusion of 20 μM Icilin and 1 μM TG in the absence of extracellular Ca^2+^.

Whole cell currents were elicited by a 100 ms linear ramp from -100 mV to +100 mV from a holding potential of -40 mV (Fig. 2A, insert: upper panel), and were recorded at steady state 2 ± 1 minutes after the patch rupture. After 3 ± 1 minutes, cells were perfused with a solution containing 20 μM of Icilin, the potent activator of TRPM8. Icilin activated a "noisy" non-selective current with a strong outward rectification (Fig. 2A). The current-density recorded in the presence of Icilin in the bath was markedly higher than before Icilin perfusion (43.42 ± 12.60 pA/pF vs 5.94 ± 1.77 pA/pF at +100 mV). The reversal potential was 8.29 ± 4.30 mV in the presence of Icilin. However, Icilin induced a response in only 4/14 cells (28.6% of all the cells tested). Fig. 2A (insert, lower panel) shows the Icilin-activated current which is calculated by substracting the control current from the current recorded after Icilin application.

To further characterize TRPM8 channels in MCF-7 cells, calcium imaging experiments were performed. Perfusion of Icilin at a concentration of 20 μM, in an external solution containing 5 mM Ca^2+ ^caused a sustained elevation of cytoplasmic calcium concentration [Ca^2+^]_c _(Fig. 2B). The proportion of responsive cells was only 32.16% of cells tested (64/199 of all the cells tested). TRPM8 protein can be present in the plasma membrane and in the endoplasmic reticulum of human prostate LNCaP cancer cell line [20]. Moreover, high concentrations of menthol in the absence of extracellular Ca^2+ ^caused a small but significant increase in intracellular Ca^2+ ^in LNCaP cells [20]. To test whether TRPM8 channels are functional at endoplasmic reticulum (ER), cells were perfused with 20 μM Icilin in the absence of external Ca^2+^. In this condition, Icilin had no effect, while application of the most potent selective SERCA inhibitor Thapsigargin (1 μM), which is often used to induce Ca^2+ ^release from endoplasmic reticulum, increased intracellular Ca^2+ ^in MCF-7 cells (Fig. 2C, n = 20). Taken together, these results show that MCF-7 cells express functional TRPM8 channels at the plasma membrane, and not at the endoplasmic reticulum membrane.

### 17-β estradiol enhanced TRPM8 expression in a steroid-deprived MCF-7 cells

**Figure 3:**
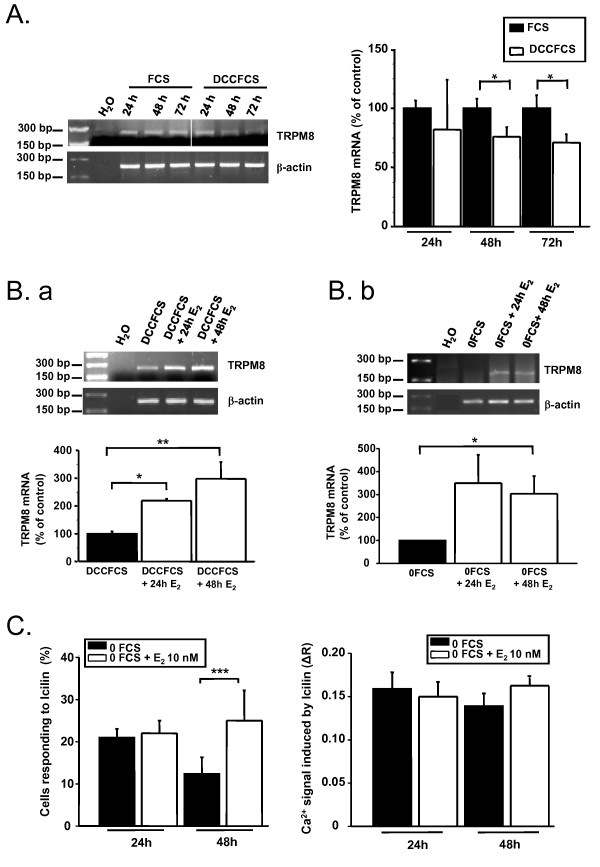
**TRPM8 mRNA expression is under hormonal control. **(A) Upper panel: agarose gel showing TRPM8 mRNA expression in MCF-7 cells which were incubated in steroid-free FCS medium (DCCFCS) for 24, 48 and 72 h. Lower panel: histogram showing a decrease of TRPM8 mRNA expression in DCCFCS medium (*, p < 0.05, three independent experiments). (B. a) Upper panel: a representative experiment showing TRPM8 mRNA expression in MCF-7 cells which were incubated in DCCFCS medium for 48 h prior to E_2 _(10 nM) application for 24 and 48 h. Lower panel: histogram showing that E_2 _increased TRPM8 mRNA expression (*, p < 0.05, **, p < 0.01, four independent experiments). (B. b) Upper panel shows an agarose gel representative of the effect of E_2 _on TRPM8 expression. MCF-7 cells were growing in 0FCS medium for 48 h prior to added E_2 _(10 nM) for 24 h and 48 h. Lower panel: histogram showing that E_2 _increased TRPM8 mRNA levels (*, p < 0.05, three independent experiments). TRPM8 mRNA levels are expressed as the ratio of TRPM8 on β-actin referent gene. (C) Histograms showing the average of cells sensitive to Icilin (left panel) and the average of the amplitude of Icilin-induced Ca^2+ ^entry (right panel) in MCF-7 cells -incubated in 0FCS for 48 h prior to E_2 _(10 nM) application for 24 and 48 h (***, p < 0.001, three independent experiments).

In order to determine whether the human TRPM8 was regulated by estrogens, we cultured MCF-7 cells in starved medium (0 FCS) or steroid-free medium (5% dextran-coated charcoal-treated FCS (DCCFCS). Incubation of MCF-7 cells in DCCFCS induced a significant decrease in TRPM8 mRNA level by 76 ± 8.4% of the control for 48 h and 71.1 ± 6.8% of the control for 72 h (p < 0.01, Fig. 3A). As 17-β estradiol (E_2_) is known to be the main physiological steroid in the breast, we studied the effects of adding E_2 _on TRPM8 expression in MCF-7 cells cultured under steroid-free conditions. After incubation in DCCFCS medium for 48 h, MCF-7 cells were stimulated with E_2 _(10 nM) for 24 h and 48 h. TRPM8 mRNA expression increased after 24 h (219.4 ± 6.7% of control, p < 0.05) and 48 h (298.3 ± 30.2% of control, p < 0.01) of incubation with E_2 _(Fig. 3Ba). We observed similar results using 0FCS medium with an increase of incubation with E_2 _after 24 h (350.1 ± 123.3% of control) and 48 h (303.6 ± 76.7% of control, p < 0.05) (Fig. 3Bb).

We then investigated the consequences of the E_2 _treatment on the Ca^2+ ^signal induced by Icilin. Fig. 3C showed that after 48 h starvation, stimulation of MCF-7 cells with E_2 _(10 nM) for 24 h failed to affect both the number of cells responding to Icilin (48/205 in 0FCS vs. 66/299 in 0FCS+E_2_), and the amplitude of Ca^2+ ^entry induced by Icilin (ΔR = 0.16 ± 0.02, n = 48 in 0FCS vs. ΔR = 0.15 ± 0.02, n = 66 in 0FCS+E_2_). However, E_2 _treatment for 48 h increased the number of cells which respond to Icilin (47/388 in 0FCS vs. 103/437 in 0FCS+E_2_, p < 0.001, Fig. 3C), without a significant change in the amount of Icilin-induced Ca^2+ ^entry (ΔR = 0.14 ± 0.01, n = 47 in 0FCS vs. ΔR = 0.16 ± 0.01, n = 103 in 0FCS+E_2_, Fig. 3C).

### TRPM8 expression is regulated by ERα in human MCF-7 cells and correlated with ER status in breast adenocarcinomas

**Figure 4:**
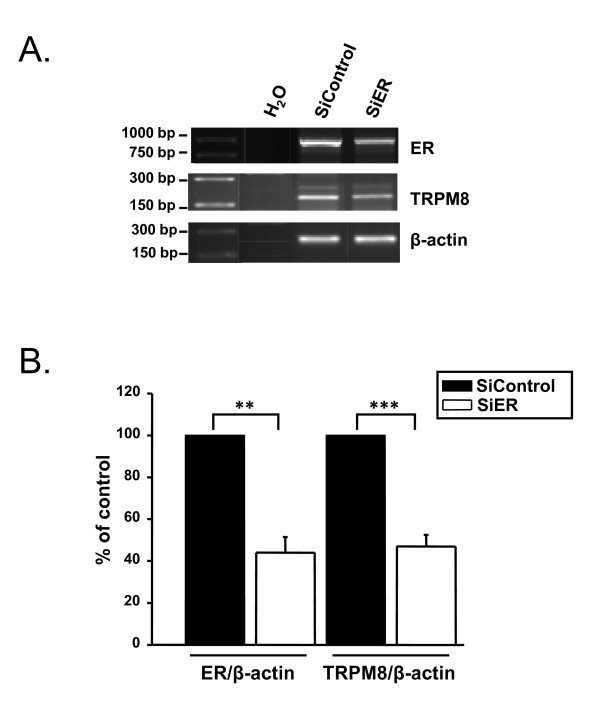
**Silencing ERα reduced TRPM8 mRNA expression. **(A) Agarose gel showing ERα and TRPM8 mRNAs expression in MCF-7 cells 72 h after transfection with SiERα. (B) Histogram showing that ERα silencing reduced TRPM8 mRNA expression (**, p < 0.01, ***, p < 0.001, three independent experiments). TRPM8 and ERα mRNA levels are expressed as the ratio of TRPM8 or ERα on β-actin referent gene.

The dependence of TRPM8 expression on the presence of ERα was studied using small interfering RNA to knockdown ERα. After siRNA-ERα transfection, MCF-7 cells were cultured in complete medium for 72 h. RT-PCR experiments confirmed that ERα was reduced by 44 ± 7.5% of the control (p < 0.01) and that TRPM8 mRNA expression decreased by 47 ± 5.5% of the control (p < 0.001) (Fig. 4).

**Figure 5:**
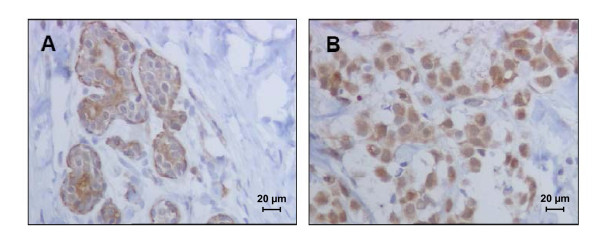
**Over-expression of TRPM8 in breast adenocarcinomas. **Immuno-histochemical staining using a specific anti-TRPM8 antibody in non tumoral (A) and breast cancer tissues (B). (Scale bar: 20 μm).

We then examined the expression of TRPM8 channels in human tumoral and adjacent non-tumoral breast tissues. Immuno-histochemical study showed a stronger cytosolic TRPM8 staining in cancerous epithelial cells (Fig. 5B) than in their non-tumoral counterpart (Fig. 5A). Statistical analysis revealed that the over-expression of TRPM8 in tumoral tissues was observed in 65.4% of the 26 ductal adenocarcinomas tested. To confirm ERα regulation of TRPM8 in a physiological context, we studied the correlation between the over-expression of TRPM8 and ERα expression on breast cancer tissues samples. We found that TRPM8 over-expression was observed in 77.8% of the ERα positive tumours and in 37.5% of the ERα negative tumours (p < 0.05, Table 1), suggesting an association between TRPM8 expression and ER^+ ^status. Because one of the parameters used in the SBR grading system is the cell differentiation, we investigated whether the TRPM8 expression varied with tumour grades. Our results show that in the ER^+ ^adenocarcinomas which over-expressed TRPM8, 42.8% (6/14) were grade I, 42.8% (6/14) were grade II and 14.2% (2/14) were grade III. These results suggest that the TRPM8 expression depending on the differentiation status.

**Table 1:** Correlation of TRPM8 over-expression with the ERα expression.

	n	Over-expression (%)	χ^2^
**TRPM8**			
ER-	8	37.5%	
ER+	18	77.8%	**0.0463**

The percentage of cases presenting an over-expression of TRPM8 according to the ERα expression was reported. A significant correlation between TRPM8 expression and ER^+ ^status was found (* p < 0.05).

## Discussion

In this study, we demonstrate that: 1) MCF-7 breast cancer cell line expressed a classical Icilin-sensitive channel, 2) Icilin induced also an increase of intracellular Ca^2+ ^that was mediated by endogenous plasma membrane TRPM8 activation, 3) only 30% of cells under investigation respond to Icilin, 4) expression of TRPM8 is regulated by estrogens, and 5) the over-expression of TRPM8 in breast adenocarcinomas is correlated with ER^+ ^status.

Functional TRPM8 channels have been clearly characterized in over-expression systems [18,19], in human cancer epithelial LNCaP cell line [20], and in primary culture of prostate epithelium cancer (PrPCa) cells [27]. Both cold and Menthol activated an inward current in LNCaP cell line [28], while Icilin activated a classical outwardly rectified TRPM8-current in PrPCa cells [22,27]. In MCF-7 cells, application of the super-cooling agent Icilin, which is ~200-fold more potent than menthol, activated both an outwardly rectifying current and an elevation of cytoplasmic Ca^2+ ^in a small fraction of cells (30% of all the cells tested). In cells responding to Icilin, the activated currents were characterized by an outward rectification and a reversal potential close to 0 mV as described in the literature for plasma membrane TRPM8 evoked currents [22,27]. The small proportion of responding cells suggest that TRPM8 channels are not widely functionally expressed in the MCF-7 breast cancer line.

In LNCaP cells, two studies have reported dual localization of TRPM8 channels in the plasma membrane (_PM_TRPM8) and endoplasmic reticulum (_ER_TRPM8). Prevarskaya's group has demonstrated that the _ER_TRPM8 is a truncated TRPM8 isoform which acts as an endoplasmic reticulum calcium releasing channel and which is not regulated by differentiation status [27,29]. In contrast, _PM_TRPM8 channels are regulated by the differentiation and androgen receptor status [27,29]. Indeed, _PM_TRPM8 is fully expressed and functional in the early stages of well-differentiated androgen-dependent prostate cancer, and disappeared in metastasis profile when androgen receptors down-regulate. MCF-7 cell line was isolated from a non invasive adenocarcinoma, and retains several characteristics of differentiated mammary epithelium including the sensibility to estradiol via estrogen receptors and the capability of forming domes [30]. Moreover, when analysing the expression of TRPM8 according to breast cancer grades, we found that TRPM8 is rather over-expressed in grade I (well differentiated) and II (moderately differentiated) than in grade III (poorly differentiated). Taken together, we can suggest that in breast cancer, TRPM8 is functional at the plasma membrane and expressed in the early primitive breast cancers presenting a well-differentiated status.

Several studies have provided evidence of a pronounced TRPM8 expression in human tumours including prostate cancer, melanoma, lung cancer, colorectal adenocarcinoma and breast cancer [17,22,31]. Our results show, for the first time, that TRPM8 protein is over-expressed in human breast adenocarcinomas and that this over-expression is specifically correlated with ERα expression. Furthermore, in ER^+ ^MCF-7 cell line, TRPM8 expression is regulated by estrogens. Indeed, either ER expression silencing or E_2 _deprivation led to a reduction in TRPM8 mRNA expression, and application of E_2 _increased TRPM8 mRNA. Moreover, stimulation of starved cells with E_2 _increased the number of cells responding to Icilin without altering the amount of Icilin-induced Ca^2+ ^entry, suggesting that estrogens control the fraction of cells expressing TRPM8 channels rather than the amount of TRPM8 channel per cell. Taken together, these results suggest a hormonal-dependent regulation of TRPM8 expression in breast cancer. Regulation of TRPM8 by androgens in prostate cancer cells was suggested by Tsavaler *et al *[17] and confirmed in LNCaP cell line [20,22]. In LNCaP cells, the analysis of the TRPM8 gene resulted in the detection of 10 putative androgen responsive elements, one in the promoter region and the others in introns of the gene [20,22]. Altogether, we can suggest that TRPM8 gene expression is under steroid hormones regulation. However, in breast cancer, the regulation of TRPM8 gene expression by putative estrogen response elements needs further investigations.

## Conclusion

In conclusion, we have shown that TRPM8 is expressed and functional in breast cancer MCF-7 cell line. Furthermore, we have provided evidence of ER mediated increase in TRPM8 mRNA expression. Finally, we have found a correlation between TRPM8 expression in tumour tissues and ERα expression.

## Competing interests

The authors declare that they have no competing interests.

## Authors' contributions

DC did the studies on the regulation of TRPM8 by estrogens (E2 and siRNA). AG and MG did the electrophysiological and the calcium imaging studies. ID-D did the immuno-histochemistry (IHC), western blot, IP, and the conventional PCR studies. HS provided us with the human biopsies and allowed us to do the IHC in his laboratory. HO-A designed the studies and wrote the manuscript. All authors have read and approved the final manuscript.

## Pre-publication history

The pre-publication history for this paper can be accessed here:

http://www.biomedcentral.com/1471-2407/10/212/prepub
